# Drug-Induced Anaphylaxis in Children

**DOI:** 10.3390/biomedicines12030527

**Published:** 2024-02-27

**Authors:** Annamaria Bianchi, Rocco Valluzzi, Giuseppe Crisafulli, Paolo Bottau, Silvia Caimmi, Fabrizio Franceschini, Lucia Liotti, Francesca Mori, Sara Riscassi, Francesca Saretta, Sara Scavone, Carlo Caffarelli

**Affiliations:** 1 UOC Pediatria, Azienda Ospedaliera San Camillo Forlanini, 00152 Roma, Italy; 2 Translational Research in Pediatric Specialties Area, Division of Allergy, Bambino Gesù Children’s Hospital, IRCCS, 00152 Roma, Italy; 3 UOC di Pediatria, Dipartimento Materno-Infantile, Università di Messina, 98100 Messina, Italy; 4 UOC di Pediatria e Neonatologia, Ospedale di Imola (BO), 40026 Imola, Italy; 5 SC di Pediatria Fondazione IRCSS Policlinico San Matteo, 27100 Pavia, Italy; 6 UOC Pediatria, Azienda Ospedaliero-Universitaria “Ospedali Riuniti”, 60123 Ancona, Italy; 7 Allergy Unit, Meyer Children’s Hospital IRCCS, 50132 Florence, Italy; 8 UOC di Pediatria Ospedale di Bolzano, Azienda Sanitaria dell’Alto Adige, 39100 Bolzano, Italy; 9 SC Pediatria, Ospedale Latisana-Palmanova, Dipartimento Materno-Infantile Azienda Sanitaria, Universitaria Friuli Centrale, 33100 Udine, Italy; 10 Clinica Pediatrica, Dipartimento Medicina e Chirurgia, University of Parma, 43126 Parma, Italy

**Keywords:** anaphylaxis, drug, children, hypersensitivity reaction, drug-induced anaphylaxis

## Abstract

Drug-induced anaphylaxis in children is less common than in adults and primarily involves beta-lactams and nonsteroidal anti-inflammatory drugs. Epidemiological studies show variable prevalence, influenced by age, gender, and atopic diseases. The pathophysiology includes IgE-mediated reactions and non-IgE mechanisms, like cytokine release reactions. We address drug-induced anaphylaxis in children, focusing on antibiotics, nonsteroidal anti-inflammatory drugs, neuromuscular blocking agents, and monoclonal antibodies. Diagnosis combines clinical criteria with in vitro, in vivo, and drug provocation tests. The immediate management of acute anaphylaxis primarily involves the use of adrenaline, coupled with long-term strategies, such as allergen avoidance and patient education. Desensitization protocols are crucial for children allergic to essential medications, particularly antibiotics and chemotherapy agents.

## 1. Introduction

Anaphylaxis is a severe, acute, life-threatening multi-system reaction associated with the release of several mediators from mast cells, inducing serious respiratory, cardiovascular, and mucocutaneous manifestations that can cause fatalities. Typical symptoms are flushing, pruritus, hives, angioedema, shortness of breath, wheezing, nausea, vomiting, diarrhea, hypotension, oxygen desaturation, and cardiovascular collapse. The disorder requires immediate medical intervention [1]. Food, drugs, and insect stings are the main triggers at all ages. However, there are differences between adulthood and childhood regarding the relative proportion of the eliciting triggers, clinical presentation, and severity of anaphylaxis [2,3,4]. In contrast to data on adults, data on pediatric drug-induced anaphylaxis (DIA) are scarcely reported. Evidence is mostly based on case series, which often include adult populations [5]. This paper aims to provide an updated review on the epidemiology, risk factors, pathophysiological mechanisms, diagnosis, and management of DIA in children, above all focusing on antibiotics, nonsteroidal anti-inflammatory drugs (NSAIDs), neuromuscular blocking agents (NMBAs), and monoclonal antibodies (mAbs).

## 2. Mechanisms of Drug-Induced Anaphylaxis

In 2017, the EAACI PRACTAL Consensus provided a better understanding of the underlying mechanisms of DIA, describing the terms “phenotype” and “endotype”. Phenotypes are defined by the clinical presentation, while endotypes refer to the cellular and molecular mechanisms underlying hypersensitivity reactions (HRs), which are characterized by diagnostic biomarkers (e.g., skin testing, serum tryptase, IgE, interleukin-6 [IL-6]) [6]. DIA is primarily an IgE-mediated reaction (type I) according to the Gell and Coombs classification [7]. Type I reactions are defined as immediate reactions mediated by IgE antibodies leading to the degranulation of mast cells and basophils with the release of proinflammatory mediators such as tryptase, histamine, leukotrienes, prostaglandins, and platelet-activating factor (PAF). The symptoms range from urticaria to anaphylaxis. Biomarkers of these reactions are serum tryptase and skin tests. IgE-mediated allergic reactions occur in individuals who have been sensitized to the culprit drug (or cross-reactive substance). Sensitization is the result of a previous asymptomatic exposure to the culprit agent [8]. Beta-lactams are common antibiotics inducing IgE-mediated DIA. There are further DIA reactions that involve non-IgE-mediated mechanisms, including non-IgE mast cell/basophil degranulation and cytokine release, as well as non-immunologic mechanisms (cyclooxygenase (COX) inhibition). For instance, mast-cell-specific receptors such as the Mas-related G-protein-coupled receptor X2 (MRGPRX2) are targets for direct activation by NMBAs (e.g., rocuronium, mivacurium, atracurium) and quinolones (e.g., ciprofloxacin) [8,9]. GPCRs (G-protein-coupled receptors) play a critical role in modulating mast cell function, as they are involved in both modifying FcεRI-mediated mast cell activation and inducing mast cell mediator release. These receptors, which include the receptor for the complement component C3a, are linked to specific G proteins, highlighting the intricate interplay between receptor interactions, G protein activation, and subsequent cellular responses [10]. 

In addition, contrast media, drug excipients, iron infusions, and vaccines can induce HRs by activating mast cells through complement receptors and C3a and C5a anaphylotoxins [8,11]. Complement activation-related pseudo-allergy (CARPA) is a non-IgE-mediated pseudo-allergy with symptoms such as flushing, hives, hypoxia, vasodilation, hypotension, and anaphylaxis [8]. Cytokine release reactions (CRRs) are typically induced by chemotherapeutic agents and monoclonal antibodies (mAbs) and mediated by T cells, monocytes and macrophages, natural killer cells, and endothelial cells [12,13]. The massive release of cytokines, including IL-6, IL-1β, and tumor necrosis factor-alpha (TNF-α), causes a wide range of symptoms, from mild flu-like symptoms to severe life-threatening manifestations. Mild symptoms of CRRs include fever, fatigue, headache, rash, arthralgia, and myalgia. Severe cases are characterized by hypotension and high fever and can progress to an uncontrolled systemic inflammatory response with vasopressor-requiring circulatory shock, vascular leakage, disseminated intravascular coagulation, and multi-organ system failure. CRRs may develop during the first drug contact, but they have also been described after several exposures. IL-6 is considered a biomarker for this type of reaction [12,13]. At last, NSAIDs can induce anaphylaxis through an enzymatic mechanism (pseudo-allergic reaction) related to the inhibition of cyclooxygenase-1, blocking prostaglandin production and increasing leukotriene production [14].

**Figure 1:**
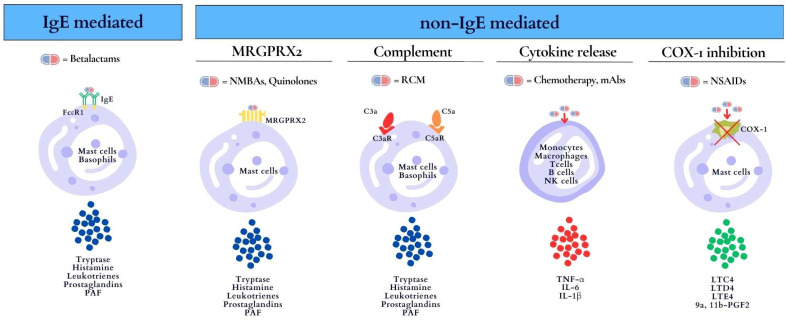
Mechanisms of DIA (drug-induced anaphylaxis) indicating culprit drugs, cells involved, and biomarkers. C3aR, C3a receptor; C5aR, C5a receptor; COX-1, cyclooxygenase-1; FcεRI, high-affinity Fc receptor for immunoglobulin E; IgE, immunoglobulin E; IL-6, interleukin-6; IL-β1, interleukin- β1; LTC4, leukotriene C4; LTD4, leukotriene D4; LTE4, leukotriene E4; mABs, monoclonal antibodies; MRGPRX2, Mas-related G-protein-coupled receptor member X2; NMBAs, neuromuscular blocking agents; NSAIDs, nonsteroidal anti-inflammatory drugs (NSAIDs); PAF, platelet-activating factor; RCM, radio contrast media; TNF-α, tumor necrosis factor-α; 9α,11β-PGF2, 9α,11β-prostaglandin F2.

The mechanisms of DIA indicating the culprit drugs, cells involved, and biomarkers are shown in Figure 1. 

## 3. Diagnosis

The recognition of DIA is based primarily on clinical criteria, including the rapid onset of multiple symptoms, as suggested by the National Institute of Allergy and Infectious Disease and the Food Allergy and Anaphylaxis Network (NIAID/FAAN) in 2006 (Table 1) [15]. The World Allergy Organization (WAO) Anaphylaxis Committee has recently updated the diagnostic criteria for NIAID/FAAN (Table 1). To simplify the existing criteria, the WAO Anaphylaxis Committee criteria has joined NIAID/FAAN criterium 1 with criterium 2. Criterium 3 was modified by adding isolated bronchospasm or laryngeal involvement [16]. From the initial descriptions based on clinical criteria, such as acute onset with the involvement of two or more organ systems or associated hypotension or upper airway compromise, the anaphylaxis definition has evolved into the identification of phenotypes and underlying endotypes supported by diagnostic biomarkers [6,15]. Recent findings suggest that fever, myalgia, arthralgia, and headache may also indicate CRRs during treatment with chemotherapeutic agents and mAbs [17]. The anaphylaxis severity is variable, and it can be classified with a severity grading according to the Ring and Messmer criteria (1997) or Brown’s criteria (2004) (Table 2) [18,19]. Both scales are currently used to classify DIA. Brown’s classification is used to categorize immediate anaphylactic reactions to chemotherapeutic agents and mAbs [20]. In DIA, skin manifestations were more common in children than in adults (81% vs. 51%) [21,22]. It is unclear whether cardiovascular symptoms, respiratory symptoms, and shock are less frequent in children than in adults or in the elderly [21,22,23]. The frequency of gastrointestinal and neurological symptoms did not differ between children and adults [22,23,24]. Among children with DIA, cyanosis was more common in children < 5 years of age, neurological symptoms in school-age children, and hypotension in adolescents [21].

The differential diagnosis of DIA includes the following manifestations: Immunization Stress-Related Responses due to “anxiety” about the immunization, in which subjective symptoms often predominate in the absence of objective ones; Oculo-Respiratory Syndrome, which has been described after influenza vaccine administration and is characterized by bilateral conjunctivitis, facial edema, and upper respiratory symptoms [25,26]. Levels of serum tryptase > [(1.2 × baseline tryptase) + 2 μg/L], 30 min to 2 hours after the onset of the reaction, are helpful for the diagnosis of DIA [1]. The lack of an elevated tryptase level does not rule out anaphylaxis, because mechanisms other than mast cell degranulation may be involved in DIA, such as in CRRs [1]. 

## 4. In Vitro Test and In Vivo Test 

Drug HRs in children present significant health concerns. Limited epidemiologic data and scarce studies on the diagnostic work-up contribute to the gaps in understanding and effectively managing these reactions [27]. Diagnosing drug allergies in children frequently relies on reports from patients or caregivers, rather than on standardized tests [28]. For children who exhibit immediate drug reactions, a combination of in vitro, in vivo, and drug provocation tests (DPTs) should ensure a confident diagnosis and proper management. Both in vitro exams, such as specific IgE testing, and in vivo tests, such as skin prick or intradermal tests, identify drug-specific antibodies. They have different sensitivities depending on the active principle and the formulation used for testing. DPTs, under medical monitoring, can confirm or rule out drug allergies. The European Network for Drug Allergy/European Academy of Allergy and Clinical Immunology (ENDA/EAACI) guidelines advise conducting in vitro or in vivo tests 4 to 6 weeks after the reaction to await acute symptoms’ resolution and reduce false-negative results [29]. When parents do not consent to in vivo tests, including DPTs, in vitro tests represent a less risky method to assess drug allergies in children [30]. In vitro tests may be considered, particularly for those with a history of severe anaphylaxis, because of the risk of reproducing systemic reactions with in vivo tests [31]. Drug-specific immunoglobulin E (IgE) is used as an in vitro tool, especially for beta-lactams and perioperative drugs such as NMBAs or chlorhexidine. The fluorimetric enzyme immunoassay (FEIA) is a commercial method by ThermoFisher, Uppsala, Sweden. Unfortunately, this immunoassay is available only for a limited group of drugs and not licensed for clinical practice in all countries (Table 3). Diagnostic tools such as Radioimmunoassays (RIAs), enzyme immunoassays (ELISAs), chemiluminescent immunoassays (CLIA, Immulite 2000 XPi Immunoassay System), or diverse solid phases (cellulose, Sepharose) are also available. Another in vitro test is the basophil activation test (BAT), in which basophils are triggered by the suspected drug, and with activation, they express CD63 or CD203c markers on their surfaces [32]. While the BAT may have limited availability in pediatric care, it is indeed a well-recognized diagnostic tool [27]. While in vitro tests offer insights into immune cell activation by drugs, their role in pediatric drug hypersensitivity is debated. The existing data are inconsistent, often coming from case reports or minor case series with divergent results [33]. The skin prick and intradermal test procedures are standardized for both children and adults. Skin prick tests typically use commercial drug preparations, often undiluted [34]. In drug shortages, a prick-to-prick test with a leftover drug solution can be used [35]. Skin prick tests with additives require proper dilutions [36]. Intradermal tests at immediate reading are performed when skin prick test results are negative in immediate HRs. The testing stops upon a positive skin test result. For severe anaphylactic cases, the starting skin test concentration should be reduced to avoid systemic reactions [37]. For low-risk patients, skin prick tests and intradermal tests can be directly performed with the highest nonirritating concentrations. In Table 4, the recommended concentrations for skin tests are reported according to Barbaud and Romano [34]. These are used to test immediate HRs and are derived from varied intradermal test methods. However, in children, these tests lack strong evidence, especially because intradermal tests are rarely assessed in younger age groups compared to adults [35]. The sensitivity of in vitro and in vivo tests varies depending on the tested medication (Table 5). A DPT is used to confirm or rule out cases of immediate drug-induced hypersensitivity. As the gold standard test, the DPT is suggested for patients with a low likelihood of a drug allergy and/or inconclusive tests, but it is not suitable for those with a clear history of severe reactions or near-fatal anaphylaxis [5]. For these individuals, it is mandatory to find safe drug alternatives, which should be tested by the DPT. There is a lack of DPT protocols specifically designed for children [27]. Both skin tests and DPTs should be performed in controlled settings. For those with a history of anaphylactic events, testing should be closely monitored in hospital settings to ensure patient safety [5].

## 5. Epidemiology

The lifetime prevalence of anaphylaxis varies widely across studies, ranging between 0.3 and 5.1%, with an incidence of 6–112 cases per 100,000 person-years [42]. Death occurs in approximately 0.3–2.2% of anaphylaxis cases [42,43]. In the general population, the incidence of fatal anaphylaxis is reported to have increased in Australia by 6% per year from 1997 to 2013 [44] and has been stable in the UK and US [45]. Drugs are the leading lifetime cause of anaphylaxis mortality in Europe, Australia, and the United States [44,45,46,47,48]. DIA deaths increased both in Australia, where it increased by 5.6% per year (1997 to 2013) [44], and in the US, where it increased from 0.27 per million in 1999–2001 to 0.51 per million in 2008–2010 [45]. In contrast, in France, anaphylaxis mortality declined for all causes by 2% per year [47], and food was a more common elicitor than drugs in the period 2002–2020 [49]. Regarding pediatric age, a systematic review by Wang et al. reported a worldwide prevalence of anaphylaxis ranging from 0.04% to 1.8% with an incidence rate ranging from 1 to 761 cases per 100,000 person-years with an increasing trend [50]. In pediatric anaphylaxis, mortality ranges from 1.4% to 2.4% [47,51]. In France, it has been reported to be lower than in adults [47]. Several studies have reported that food is the most frequent trigger of anaphylaxis in children, followed by drugs or insect venom [48,50,52]. The worldwide incidence of food-induced anaphylaxis ranged from 1 to 77 per 100,000 person-years, followed by drugs (from 0.3 to 10.6) and by insect venom (from 0.02 to 8.7) in childhood [50]. Data collected by the European Anaphylaxis Registry in 2016 showed that 1291 (66%) out of 1970 cases of children with anaphylaxis were caused by food items, 381 (19%) cases were due to insect venom, and 101 (5%) cases were provoked by drugs [52]. Similar results were reported in Turkish children [53]. In the Taiwan Emerging Department, anaphylaxis to medications occurred in 13 cases, to foods in 6 cases, and to insect venom in 0 cases [22]. DIA death in children is rarely reported [46,49]. The great variability in epidemiologic data could be related to several factors, including different diagnostic criteria, different study designs, and genetic and geographical differences. Children’s inability to describe their symptoms is one contributing factor, where up to 50% of DIA cases do not exhibit skin symptoms. The assessment of blood pressure in infants and children is also challenging, and tryptase is not commonly measured.

An Australian study on pediatric anaphylaxis showed that nearly 50% of the diagnoses are missed [54]. It should also be considered that the frequency of self-reported drug hypersensitivity reactions (HRs) is much higher than that after a proper allergy work-up, both in the general population and in childhood. The probability of a true allergy increases with the severity of the reaction. Systematic reviews and meta-analyses report self-reported prevalences of drug allergies of 10.0% in adults and 5.1% in children [55].

## 6. Risk Factors 

Several risk factors for DIA have been reported in childhood. Contrasting data have been found on the role of gender. In a European population of children and adults, DIA was more common in females, with a rate that was higher than in anaphylaxis due to other triggers [24]. However, DIA mortality was significantly higher in males both in hospital databases and in forensic series in Spain, including children and adults [48]. Data on children with DIA found that males were more frequent in China [21] and Latin America [23] and females in Turkey [53]. A North American study reported a gender shift from male to female when comparing young children to adolescents [51]. An explanation is that estradiol may increase the mast cell releasability. Regarding age, the rate of DIA in childhood is lower (8%) than in adults [56]. It may be hypothesized that a longer exposure may favor the development of sensitization. In adolescents (age range: 13–17 years), the frequency is almost doubled compared to younger ages, probably because of age-related sensitization and/or the use of different therapeutic products [57]. The likelihood of severe DIA is reported to be over 15 times lower and less severe in children aged 0 to 5 years than in children aged 13 to 17 years [21]. More severe reactions that need to be treated in intensive care units are less common in children (10.46%) than in adults (11.30%) and the elderly (14.94%) [24]. Consistently, the probability of fatal DIA was lower in children and young adults than in patients > 44 years of age [48]. A previous history of anaphylactic reactions to foods, co-existent asthma, and atopic diseases are considered the main risk factors for anaphylaxis to foods [49,58,59]. However, atopy and/or atopic diseases, including asthma and food allergies, have not been reported as significant risk factors for DIA in studies that globally enrolled 191 children [53,56,60]. However, Jares et al. [23] found that 57% of children with DIA had atopy, 53% rhinitis, and 32% asthma. A previous HR to the culprit drug is quite common in children with DIA. Taking together the results of four studies, it was reported in 53 out of 151 cases [23,52,53,60].

Mastocytosis has been reported as a risk factor for DIA, particularly in the perioperative setting. Approximately 4% of children with mastocytosis may develop systemic symptoms because of mast cell activation under different anesthetic procedures [61]. Physical or mechanical factors are at least equally significant in provoking mast cell degranulation during anesthesia. Because anaphylaxis in children with mastocytosis is most commonly idiopathic, the role of drugs is overestimated [62]. Some studies have reported a higher risk of DIA in children with systemic illnesses, most often malignancies, storage diseases, cardiovascular diseases, seizure disorders, renal diseases, coagulation abnormalities, and rheumatologic diseases, and/or taking other medications [56]. A parental route of administration [60] predisposes to DIA, as occurs in children with cystic fibrosis [27]. There was no difference among subcutaneous, intramuscular, intravenous bolus, and continuous infusion routes [21]. The risk is higher in children with more than one risk factor [3]. 

## 7. Management 

Anaphylaxis represents a critical clinical emergency that demands that all healthcare practitioners are able to recognize and effectively manage it. The EAACI Anaphylaxis Task Force has recently revised the 2014 guidelines for the management of anaphylaxis in childhood. These guidelines were developed by using a rigorous framework and approach, taking into consideration the AGREE II framework and the GRADE approach [1]. 

### 7.1. Acute Phase

During the acute phase of anaphylaxis in children, prompt management is crucial. This involves the immediate administration of adrenaline, which is the first-line treatment for anaphylaxis. The delayed or incorrect administration of adrenaline increases the risk of biphasic anaphylactic reactions and potentially fatal outcomes. In summary, the protocol should include the following key aspects:

- Recognize anaphylaxis symptoms and signs, suspend the culprit drug, and optimize patient posture.
- Immediately administer intramuscular adrenaline in the mid-thigh area as the first-line management of anaphylaxis. The EAACI Task Force suggests a dose of 0.01 mg/kg up to a maximum of 0.5 mg in healthcare settings, or 0.15 mg (for children from 7.5 kg to 25–30 kg), 0.3 mg (for children from 25 to 30 kg), and 0.5 mg (for adolescents when the patient is overweight or has experienced a previous episode of life-threatening anaphylaxis) in community settings.
- Give high-flow oxygen at 10 liters/minute if there are circulatory/severe respiratory symptoms: i.v. fluid—crystalloid bolus 10 mL per kg of patient’s weight (in children < 25–30 kg) or 500 mL (in children > 25–30 kg). If no improvement in 5–10 min is observed, repeat the adrenaline dose and give intravenous fluids.
- Call the emergency team, including critical care experts, to provide advanced treatment, including adrenaline infusion; in case of cardiac arrest, follow the guidelines.
- Monitor the cerebral status, pulse oximetry, blood pressure, and ECG.
- When the patient is stabilized, measure serum tryptase 30 min to 2 h after the reaction onset and consider additional treatment (antihistamines, corticosteroids).

### 7.2. Following the Resolution of the Acute Phase

Following the resolution of the acute phase of anaphylaxis in children, it is important to provide management and follow-up:

- Educate the child and their caregivers about anaphylaxis, including triggers, signs, and symptoms, and the importance of avoiding allergens.
- Ensure the child has access to self-injectable epinephrine and properly teach them or their caregivers how to use it.
- Develop an emergency action plan that outlines steps to take in the event of another anaphylactic reaction, including when and how to administer epinephrine and when to seek medical help.
- Ensure regular follow-up appointments with healthcare providers to monitor the child’s allergies, assess any potential triggers or changes in allergens, and adjust treatment plans accordingly.
- Assess the need for allergy testing to identify specific allergens and potential avoidance strategies.
- Provide appropriate counseling and support to help the child and their caregivers cope with the emotional and psychological impacts of living with severe allergies.
- Collaborate with personnel at school or in other educational settings to ensure a safe environment for the child, including staff training on recognizing and managing anaphylactic reactions.
- Most of the time, complete avoidance of the drug is the safest choice, and there is no need to provide injectable adrenaline as part of the emergency plan. However, for patients with DIA and cardiovascular disease, providing injectable adrenaline to parents/patients might be recommended [1,5].

## 8. Desensitization Protocols 

Desensitization protocols are essential interventions for children who exhibit allergies to necessary medications. When alternative drugs prove to be less effective, desensitization can temporarily enhance tolerance, facilitating the safe reintroduction of the drug. Desensitization protocols should be performed under experienced healthcare providers’ supervision in a controlled setting, such as a specialist allergy center with personnel trained in the management of drug hypersensitivity in children. The decision to proceed with desensitization should consider the potential risks and benefits for the individual child, as well as his/her medical history and current health status. Desensitization protocols in the pediatric population have mainly been studied for antibiotics, such as beta-lactams, and chemotherapy for young children with solid tumors [63]. In particular, children and adolescents with cystic fibrosis have a propensity for infections, most notably caused by Pseudomonas aeruginosa. They must frequently receive antipseudomonal drugs. Because of frequent and repeated exposures to antibiotics, cystic fibrosis patients have a prevalence of up to 70% of allergic reactions and a high risk for potentially life-threatening reactions to antibiotics, especially beta-lactams, with a frequent need for desensitization procedures [64,65]. There is a need for more extensive research and documentation to cover a broader range of drugs, including mAbs, which are increasingly used [63]. Desensitization protocols for rituximab, infliximab, and tocilizumab in children have been successfully described in various studies [66,67,68,69,70]. The three-bag 12-step protocol is the most widely applied to the pediatric population (Table 6), based on the one originally proposed by Castells et al., while 16-step (four bags) and 20-step (five bags) protocols are reserved for patients who experienced life-threatening anaphylaxis [71,72]. Further investigations and published procedures, particularly for pediatric patients, are necessary to cover a broader range of drugs.

## 9. Culprit Drugs

Anaphylaxis has been described as a possible adverse effect of any drug at any dose. In almost all studies, antibiotics and NSAIDs represent the major culprits of DIA in children: this is probably related to the high prescription rates of these drugs in children [2,5,52]. There are differences among countries that may be due to consumption. Data provided by tertiary referral centers specialized in pediatric allergology showed that drugs were elicitors of anaphylaxis primarily for adolescents, and analgesics and beta-lactam drugs were the most common causal agents [52]. In Canada, a study found that out of 51 patients with DIA, the highest percentage of reactions (62.7%) were caused by non-antibiotic drugs, with NSAIDs being the primary culprit (21.6%). Antibiotic-related reactions made up 37.3% of the total reactions, with beta-lactams being the most commonly suspected (31.4%) [60].

A recent Chinese retrospective study was conducted in 110 children aged 0–16 years referred to the emergency department for anaphylaxis. The triggers of anaphylaxis were drugs in 37 cases (33.6%). The most common drug triggers were antibiotics (59.4%, 22/37), NSAIDs (10.8%, 4/37), vaccines (10.8%, 4/37), and herbal medicines (10.8%, 4/37) [73]. 

Beta-lactams are the leading cause of DIA in children [74]. For patients who experienced anaphylactic reactions to penicillins and/or cephalosporins, the EAACI position paper recommends avoiding the entire class of beta-lactam antibiotics and referring the patient to an allergist for further evaluation. However, if an allergy evaluation is not feasible and beta-lactam therapy is deemed necessary, it is advisable to consider a graded challenge with third-/fourth-/fifth-generation cephalosporins for those who reacted to penicillins or penicillins with side chains different from those of the culprit cephalosporins, or with carbapenems or aztreonam for those who reacted to cephalosporins. For non-beta-lactam antibiotics, few cases of anaphylaxis to macrolides have been reported in children. A Turkish study involving 61 patients with suspected allergies to macrolides, including 39 who reacted to clarithromycin and 22 to azithromycin, found that only one patient reported an anaphylactic reaction, which occurred during a DPT with azithromycin [75]. 

NSAIDs are a significant cause of pediatric DIA, with ibuprofen often leading the list. Anaphylaxis was reported with a rate between 9% and 32% in children and adolescents with proven NSAID hypersensitivity [76]. The classification of acute HRs to NSAIDs is outlined in both European and American guidelines. Anaphylactic reactions are categorized into two clinical types: NSAID-induced urticaria/angioedema or anaphylaxis (NIUAA) and single NSAID-induced urticaria/angioedema or anaphylaxis (SNIUAA) [77,78,79,80]. NIUAA is primarily related to cyclooxygenase-1 (COX-1) inhibition, leading to a cross-reactivity pattern with multiple NSAIDs that inhibit COX-1 [77,81,82,83]. In SNIUAA, an IgE-mediated mechanism may be involved [77,78]. The gold standard for diagnosing NSAID hypersensitivity in children is the DPT [77,84], while there is no indication to perform skin tests in those with NIUAA [77]. Desensitization is usually discouraged in pediatric cases, and the choice of alternative NSAIDs is preferred. If no alternatives are available, desensitization might be considered, particularly for patients with specific medical conditions [84,85]. NSAIDs can act both as a cofactor in the induction of food allergies (NIFA) and as a factor in their exacerbation (NEFA), as shown in a population of both adolescent and adult patients [86]. The prevalence of anaphylactic reactions differs across distinctive settings, including the perioperative context, where NMBAs are a notable trigger, despite geographical differences [87]. In France, peri-anesthetic anaphylactic reactions were estimated at 1:7741 procedures, with NMBA reactions at 1:81,275 procedures [88]. In children, the incidence is lower, ranging from 1:2100 in a French pediatric center to 1:37,000 in the NAP6 study [89,90]. NMBA-induced anaphylaxis usually presents within five minutes, with severe hypotension being the common sign in children. Approximately 4% of NMBA anaphylaxis cases result in fatalities despite treatment [91]. Non-IgE-mediated anaphylaxis is caused by direct mast cell histamine release from benzyl-isoquinolines (mivacurium, atracurium, cisatracurium), whereas IgE-mediated reactions account for approximately 60% of NMBA reactions. The interaction between NMBAs and the mast cell receptor MRGPRX2 has been proposed as a pathomechanism [92]. Succinylcholine has the highest cross-reactivity with rocuronium or vecuronium, while cisatracurium exhibits the lowest one [90,93]. Anaphylactic reactions induced by mAbs are infrequent and primarily reported in pediatric cases involving drugs such as anakinra, omalizumab, rituximab, and tocilizumab [12]. These reactions can be categorized into two main mechanisms: IgE-mediated reactions with rapid onset and reactions driven by massive cytokine release, which are more severe and life-threatening [12]. Quick-onset symptoms characterize IgE-mediated mAb anaphylactic reactions, which may even manifest in several hours if the subcutaneous route is used [12]. Diagnostic indicators include the acute elevation of serum tryptase levels and positive skin tests with a nonirritating mAb concentration [12]. Reactions resulting from a high cytokine release are classified into two endo-phenotypes: infusion-related reactions and CRRs. CRRs are more severe, are occasionally life-threatening, and require desensitization. IL-6 has been proposed as a biomarker for CRRs, with a 40-fold elevation from baseline as a potential cutoff [94]. Mixed reactions combining characteristics of both CRRs and IgE-mediated reactions have been reported, with specific biomarkers for both endotypes (elevated IL-6 and increased serum tryptase/positive skin tests) [12,94]. No specific pediatric data on this topic have been published yet. 

## 10. Conclusions

Epidemiologic data on DIA in children are incomplete. Studies on risk factors are necessary to determine the role of comorbidities, previous anaphylactic reactions, and elicitors of the reactions. DIA in children represents a significant clinical challenge. Antibiotics, particularly beta-lactams, and NSAIDs are the primary triggers, with the prevalence and severity of reactions varying with age and sensitization patterns. Integrating clinical criteria with a combination of in vitro testing, in vivo testing, and the DPT is crucial for accurately diagnosing immediate drug reactions. Management strategies must prioritize immediate intervention with epinephrine during acute episodes and comprehensive education and prevention plans for the future. Desensitization protocols, although used less frequently in children, are valuable for those allergic to necessary medications. A multidisciplinary approach involving allergists, pediatricians, and emergency care providers is essential for the optimal management and prevention of DIA in children.

## Figures and Tables

**Table 1:** NIAID/FAAN 2006 and WAO 2020 criteria for anaphylaxis.

NIAID/FAAN Criteria [15]	WAO Criteria [16]
Anaphylaxis is highly likely when any of the following three criteria are fulfilled:	Anaphylaxis is highly likely when any of the following two criteria are fulfilled:
Acute onset of an illness (minutes to several hours) with involvement of the skin, mucosal tissue, or both (i.e., generalized hives, pruritus or flushing, swollen lips–tongue–uvula) AND AT LEAST ONE OF THE FOLLOWING (a) Respiratory compromise (i.e., dyspnea, wheeze/bronchospasm, stridor, reduced peak expiratory flow, and hypoxemia) (b) Reduced BP or associated symptoms of end/organ dysfunction (i.e., hypotonia [collapse], syncope, incontinence).	Acute onset of an illness (minutes to several hours) with simultaneous involvement of the skin, mucosal tissue, or both (i.e., generalized hives, pruritus or flushing, swollen lips–tongue–uvula) AND AT LEAST ONE OF THE FOLLOWING: (a) Respiratory compromise (i.e., dyspnea, wheeze/bronchospasm, stridor, reduced peak expiratory flow, hypoxemia) (b) Reduced BP or associated symptoms of end-organ dysfunction (i.e., hypotonia [collapse], syncope, incontinence) (c) Severe gastrointestinal symptoms (i.e., severe crampy abdominal pain, repetitive vomiting), especially after exposure to non-food allergens.
2. Two or more of the following that occur rapidly after exposure to a likely allergen for that patient (minutes to several hours): (a) Involvement of the skin–mucosal tissue (i.e., generalized hives, itch/flush, swollen lips–tongue–uvula) (b) Respiratory compromise (i.e., dyspnea, wheeze/bronchospasm, stridor, reduced peak expiratory flow, hypoxemia) (c) Reduced BP or associated symptoms (i.e., hypotonia [collapse], syncope, incontinence) (d) Persistent gastrointestinal symptoms (i.e., crampy abdominal pain, vomiting).	
3. Reduced BP after exposure to known allergen for that patient (minutes to several hours): (a) Infants and children: low systolic BP (age-specific) or 30% decrease in systolic BP * (b) Adults: systolic BP of <90 mmHg or >30% decrease from that person’s baseline. * Low systolic blood pressure for children is defined as <70 mmHg from 1 month to 1 year, less than 70 mmHg + [2 × age] from 1 to 10 years, and <90 mmHg from 11 to 17 years.	2. Acute onset of hypotension ** or bronchospasm or laryngeal involvement after exposure to a known or highly probable allergen for that patient (minutes to several hours), even in the absence of typical skin involvement. ** Hypotension defined as a decrease in systolic BP greater than 30% from that person’s baseline, OR infants and children under 10 years: systolic BP less than (70 mmHg + [2 × age in years]), OR adults and children over 10 years: systolic BP less than <90 mmHg.

BP, blood pressure.

**Table 2:** Comparison of anaphylaxis severity indexes.

Symptoms	Ring and Messmer’s Severity Index [18]	Brown’s Severity Index [19]
Skin lesions	Grade 1	Grade 1
Gastrointestinal and respiratory disturbances	Grade 2	Grade 2
Non-life-threatening cardiovascular symptoms (tachycardia or hypotension)	Grade 2	Grade 3
Shock and life-threatening smooth muscle spams	Grade 3	Grade 3
Cardiac and/or respiratory arrest	Grade 4	Not applicable

**Table 3:** Drugs tested by FEIA. From Saretta et al., 2023 [30].

Specific IgE Determination by FEIA	
Antibiotics	Amoxycilloyl, ampicilloyl, penicilloyl G, penicilloyl V, cefaclor
Hormones	Human insulin; pancreatin (research only)
Gelatin	Bovine gelatin
Opioids	Pholcodine
Perioperative drugs	Morphine, chlorhexidine, suxamethonium; rocuronium (research only)

**Table 4:** Highest nonirritating concentrations recommended for antibiotic, NSAID, NMBA, and mAb prick and intradermal testing. From Barbaud and Romano [34].

	Intradermal Tests	Skin Prick Tests
Beta-Lactams		
Amoxicillin, ampicillin, and other semisynthetic penicillins	20 mg/mL	20 mg/mL
Aztreonam	2–20 mg/mL	2–20 mg/mL
Benzylpenicilloyl-poly-L-lysine	6 × 10^−5^ mol/L	6 × 10^−5^ mol/L
Benzylpenicilloyl-octa-L-lysine	8.64 × 10^−5^ mol/L	8.64 × 10^−5^ mol/L
Sodium benzylpenilloate	1.5 × 10^−3^ mol/L	1.5 × 10^−3^ mol/L
Benzylpenicillin	10,000 IU/mL	10,000 IU/mL
Cefepime	2 mg/mL	2 mg/mL
Cephalosporins other than cefepime	20 mg/mL	20 mg/mL
Clavulanic acid	20 mg/mL	20 mg/mL
Imipenem-cilastatin	0.5 mg/mL–0.5 mg/mL	0.5 mg/mL–0.5 mg/mL
Ertapenem and meropenem	1 mg/mL	1 mg/mL
Quinolones		
Ciprofloxacin	0.006 mg/mL	0.006 mg/mL
Levofloxacin	0.025 mg/mL	0.025 mg/mL
Ofloxacin	0.05 mg/mL	0.05 mg/mL
Pefloxacin	None	0.32 mg/mL
Rifampicin	2 mcg/mL	2 mcg/mL
Macrolides		
Azithromycin	0.01 mg/mL	0.01 mg/mL
Clarithromycin	0.05 mg/mL	0.05 mg/mL
Erythromycin	0.01–0.05 mg/mL	5 mg/mL
Rovamycin	37.5 IU/mL	37.5 IU/mL
Others		
Clindamycin	15 mg/mL	15 mg/mL
Cotrimoxazole	0.8 mg/mL	0.8 mg/mL
Gentamycin	4 mg/mL	4 mg/mL
Rifampicin	0.002 mg/mL	0.002 mg/mL
Tobramycin	4 mg/mL	4 mg/mL
Vancomycin	0.005–0.05 mg/mL	0.005–0.05 mg/mL
Nonsteroidal Anti-Inflammatory Drugs		
Diclofenac	2.5 mg/mL	2.5 mg/mL
Ketoprofen	2 mg/mL	2 mg/mL
Piroxicam	2 mg/mL	2 mg/mL
Pyrazolones and other injectable NSAIDs	0.1 mg/mL	0.1 mg/mL
Paracetamol/Acetaminophen	1 mg/mL	1 mg/mL
Neuromuscular Blocking Agents		
Atracurium	0.01 mg/mL	1 mg/mL
Cisatracurium	0.02 mg/mL	2 mg/mL
Mivacurium	0.002 mg/mL	0.2 mg/mL
Pancuronium	0.02 mg/mL	2 mg/mL
Rocuronium	0.05 mg/mL	10 mg/mL
Suxamethonium	0.1 mg/mL	10 mg/mL
Vecuronium	0.04 mg/mL	4 mg/mL
Monoclonal Antibodies		
Adalimumab	50 mg/mL	50 mg/mL
Etanercept	5 mg/mL	5 mg/mL
Infliximab	2 mg/mL	2 mg/mL
Infliximab	10 mg/mL	10 mg/mL
Omalizumab	1.25 mcg/mL	1.25 mcg/mL
Rituximab	10 mg/mL (7 negative controls)	10 mg/mL (7 negative controls)
Tocilizumab	0.2 mg/mL or 20 mg/mL (10 negative controls) 1.62 mg/mL	0.2 mg/mL or 20 mg/mL (10 negative controls) 1.62 mg/mL

**Table 5:** Sensitivity (%) of in vitro and in vivo tests of the most frequent drugs involved in pediatric anaphylaxis.

Culprit Drugs	Specific IgE	Basophil Activation Test	Skin Tests
Beta-lactams	0–85% [38]	44–63% [38]	70% [35]
NMBA			
- Rocuronium	74–89% [39]	73–83% [39]	92–99% [39]
NSAIDs			
- Dipyrone	nv	42–65% [38]	>90% * [40]
mAb	26–68% [38]	nv [41]	nv [41]

* In case of an anaphylactic reaction. mAb, monoclonal antibodies; NMBA, neuromuscular blocking agent; NSAIDs, nonsteroidal anti-inflammatory drugs; nv, not valuable.

**Table 6:** Desensitization protocol for rituximab in pediatric patients *. Adapted from Dilley et al., 2016 [66].

	Volume (mL)	Drug per Bag (mg)	Concentration (mg/mL)			
Solutions 1	250	2.06	0.008			
Solutions 2	250	20.6	0.082			
Solutions 3	250	205.189	0.821			
Step n.	Solution n.	Rate (ml/h)	Rate (mg/kg/h)	Time (min)	Dose per step (mg)	Cumulative dose (mg)
1	1	1	0.0006	15	0.0021	0.0021
2	1	2.5	0.002	15	0.0052	0.0073
3	1	5	0.003	15	0.0103	0.0176
4	1	10	0.006	15	0.0206	0.0382
5	2	2.5	0.02	15	0.0515	0.0897
6	2	5	0.03	15	0.103	0.1927
7	2	10	0.07	15	0.206	0.3987
8	2	20	0.1	15	0.412	0.8107
9	3	5	0.3	15	1.0259	1.8366
10	3	10	0.7	15	2.0519	3.8885
11	3	20	1.3	15	4.1038	7.9923
12	3	30	2	482.5	198.0078	206.0001
Therapeutic dose 206 mg						

* It differs from the standard adult 12-step protocol mainly because of the lower infusion rate of the last step.

## Data Availability

Data sharing is not applicable.
